# A Rare Case of Right Paraduodenal Hernia: Clinical Insights and Literature Review

**DOI:** 10.7759/cureus.116753

**Published:** 2026-09-22

**Authors:** Patrícia Bárbara, Paulo Clara, Vera Pedro, Alexandra Rocha, Ana Dias

**Affiliations:** 1 General Surgery, Unidade Local de Saúde da Região de Leiria, Leiria, PRT; 2 General and Colorectal Surgery, Unidade Local de Saúde da Região de Leiria, Leiria, PRT

**Keywords:** bowel ischemia, indocyanine green fluorescence, internal hernia, intestinal obstruction, paraduodenal hernia

## Abstract

Internal hernias, although rare, are a notable cause of small bowel obstruction, with congenital paraduodenal hernias (PDHs) accounting for approximately more than half of these cases. Right PDHs, which are less common than their left-sided counterparts, present diagnostic challenges due to their rarity and complex presentations, often involving bowel strangulation. We report the case of a 34-year-old male with a history of recurrent abdominal pain who presented with acute symptoms. Imaging revealed a right PDH containing slightly distended small bowel loops. Parietal enhancement was preserved, with no signs of ischemia. A median laparotomy was performed to reduce the hernia contents and confirm bowel viability using indocyanine green dye, followed by medial mobilization of the right colon and partial excision of the hernial sac. Recovery was uneventful, and the patient was discharged on the seventh postoperative day.

Right PDH is a rare, yet important, cause of intestinal obstruction. It is often difficult to diagnose, and prompt imaging and surgical intervention are critical to prevent serious complications. While laparotomy remains the mainstay approach, laparoscopic techniques are being preferred. Accurate diagnosis and meticulous surgical repair are essential to prevent recurrence and ensure good outcomes.

## Introduction

Internal hernias are an uncommon cause of small bowel obstruction (SBO), accounting for 0.2% to 0.9% of all SBO cases, but they carry a high risk of morbidity and mortality if not promptly recognized and treated [1-4]. These hernias occur when a bowel segment protrudes through a congenital or acquired defect in the mesentery or peritoneum. Among them, congenital paraduodenal hernias (PDHs) are the most frequent type, representing approximately 53% of all internal hernias [4].

Paraduodenal hernias are embryological anomalies resulting from incomplete midgut rotation and abnormal peritoneal fixation during fetal development [4,5]. They are anatomically classified into left and right types. Left-sided PDHs are the most common, occurring in a 3:1 ratio compared to right-sided PDHs [5]. The anatomical distinction between the two is crucial: a left PDH occurs when the bowel herniates into the fossa of Landzert, bordered anteriorly by the inferior mesenteric vein. In contrast, a right PDH involves herniation into the fossa of Waldeyer, located behind the superior mesenteric artery (SMA) and superior mesenteric vein (SMV), which lie directly along the anterior margin of the hernial sac [4-7].

Because of their rarity and complex anatomy, right PDHs pose a significant diagnostic challenge. Patients may present with atypical symptoms ranging from chronic vague abdominal pain to acute SBO. Despite their rarity, right PDHs carry a lifetime risk of incarceration or strangulation of up to 50%, with reported mortality rates ranging from 20% to 50% in acute settings [3,5,7]. Therefore, early diagnosis using imaging techniques, particularly CT, is essential to identify the anatomical defect, assess bowel viability, and guide prompt surgical intervention [2,5,8].

Here, we present the case of a 34-year-old male diagnosed with a right PDH causing intestinal obstruction. Surgical treatment was successful, emphasizing the need for early recognition and prompt surgical intervention to reduce morbidity and mortality [5,9]. This case highlights the importance of a high level of suspicion combined with a thorough diagnostic and therapeutic approach in managing right PDHs [5].

## Case presentation

A 34-year-old male presented to our emergency department with a three-hour history of acute abdominal pain. Upon admission, he was afebrile and hemodynamically stable (blood pressure 120/75 mmHg, heart rate 82 beats per minute). He reported nausea but denied vomiting or other symptoms. The patient had no history of previous abdominal surgeries. Notably, over the preceding eight months, he had experienced multiple self-resolving episodes of abdominal pain associated with obstipation and vomiting, leading to several emergency department visits. Previous gastroenterological evaluations during those episodes had not identified any gastrointestinal pathology.

On physical examination, there was pain in the right lower quadrant, with rebound tenderness and active bowel sounds. Laboratory investigations on admission revealed a hemoglobin level of 15.0 g/dL, leukocytosis, and a normal C-reactive protein (CRP) level. Arterial blood gas analysis demonstrated a respiratory alkalosis with mild hyperlactatemia (lactate 2.7 mmol/L).

An abdominal CT scan was obtained, revealing a conglomerate of distal small bowel loops located posterior to the third portion of the duodenum, which was consistent with a right paraduodenal hernia. The hernial sac measured approximately 12 x 14 x 18 cm and contained the superior mesenteric vessels along its anterior wall. Several bowel segments within the sac were slightly distended; however, parietal enhancement was preserved, suggesting viable tissue without signs of ischemia (Figures 1-2).

**Figure 1:**
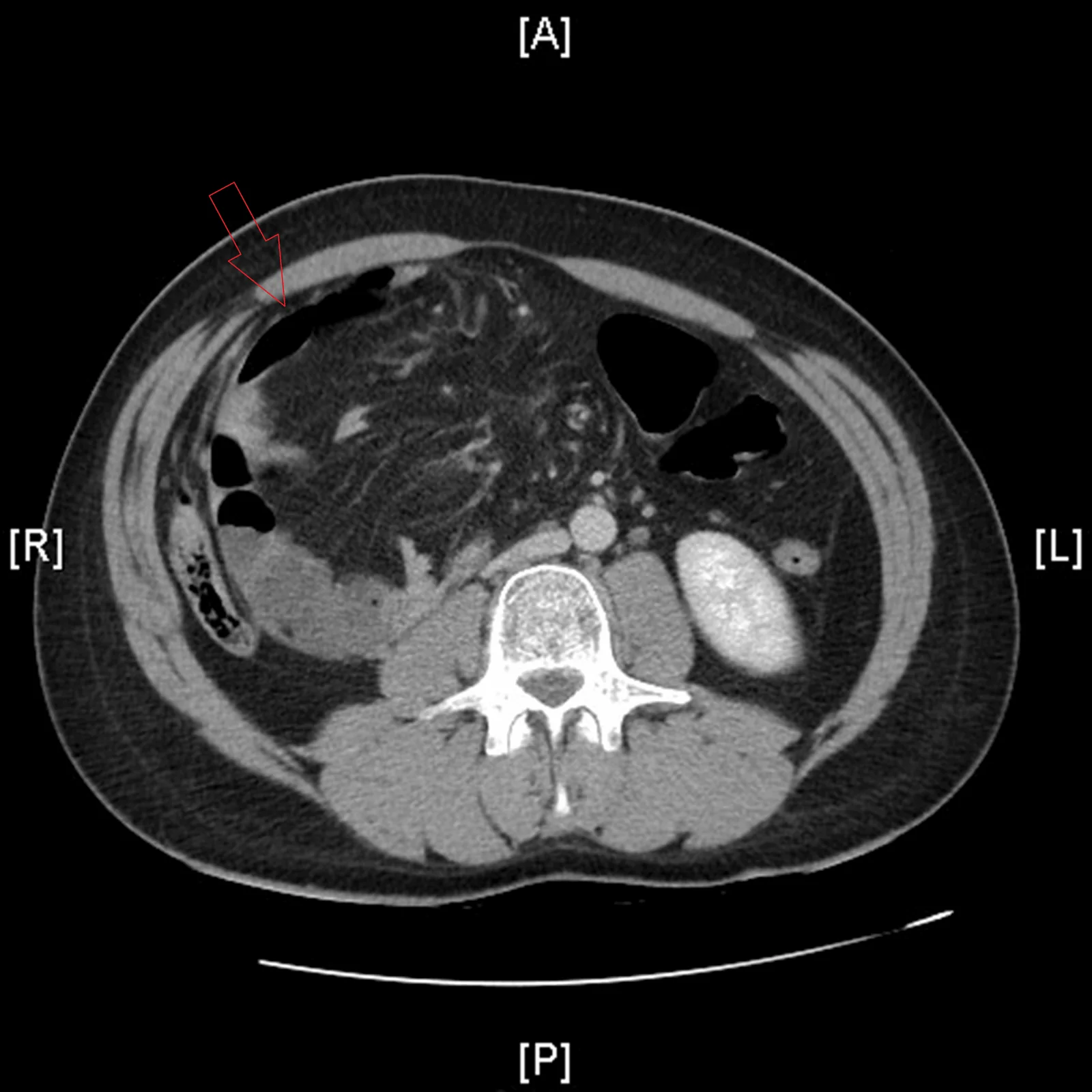
Axial view of the right PDH The CT scan with IV contrast revealed a conglomerate of distal small bowel loops located posterior to the third portion of the duodenum (red arrow), consistent with a right PDH. PDH: Paraduodenal hernia

**Figure 2:**
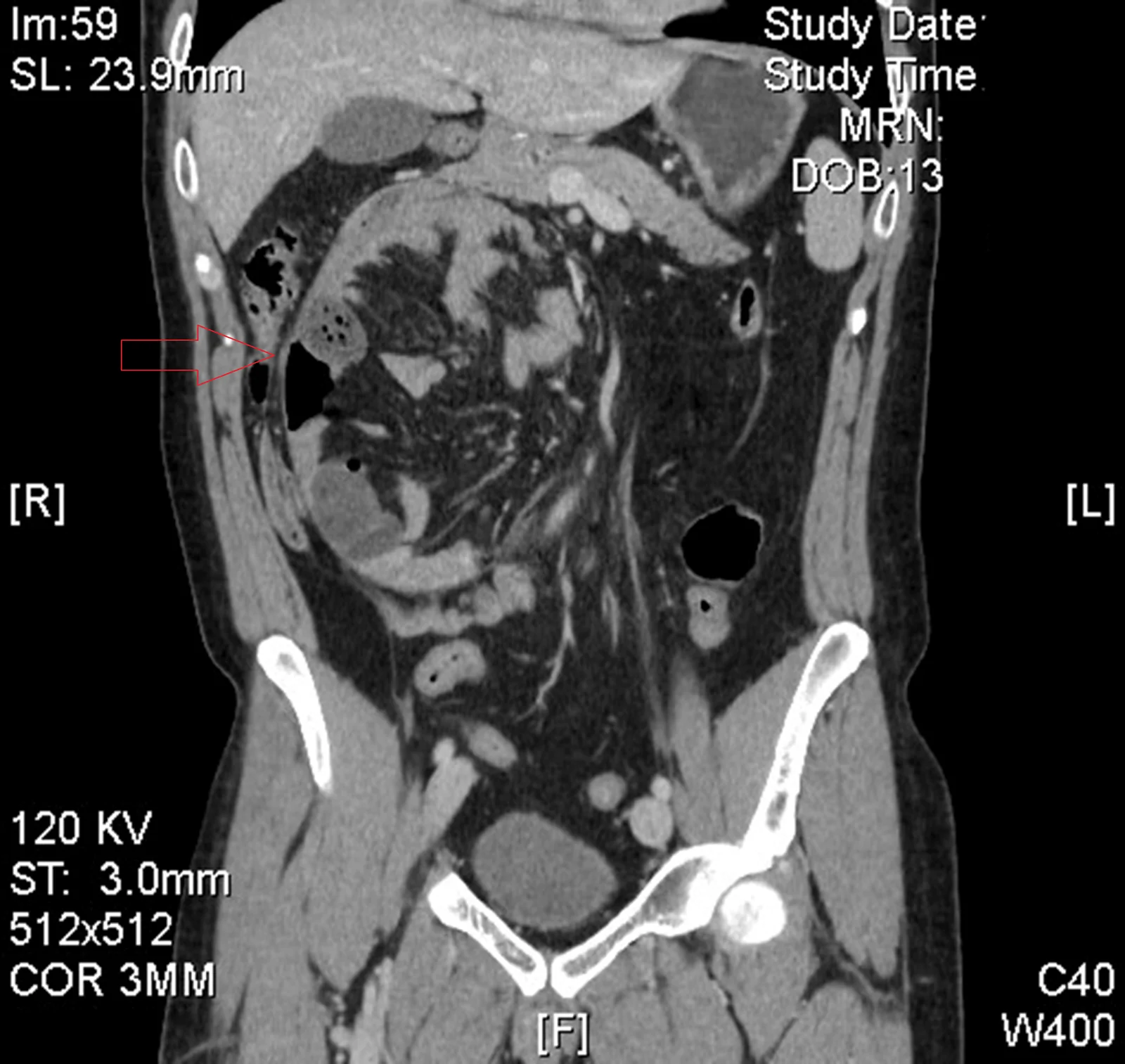
Coronal view of right PDH Seen on the CT scan with IV contrast is a conglomerate of distal small bowel loops located posterior to the third portion of the duodenum (red arrow), consistent with a right PDH. PDH: Paraduodenal hernia

Based on these findings, the patient was taken to the operating room, and a median laparotomy was performed. Intraoperatively, approximately 1.5 m of the small bowel was found incarcerated within the hernial sac. After the reduction of the hernia contents (Figure 3), formal primary closure of the mesenteric defect was not attempted due to the high risk of iatrogenic injury to the superior mesenteric vessels located along its anterior margin. Instead, medial mobilization of the right colon up to the transverse colon and a partial excision of the hernial sac were performed (Figure 4).

**Figure 3:**
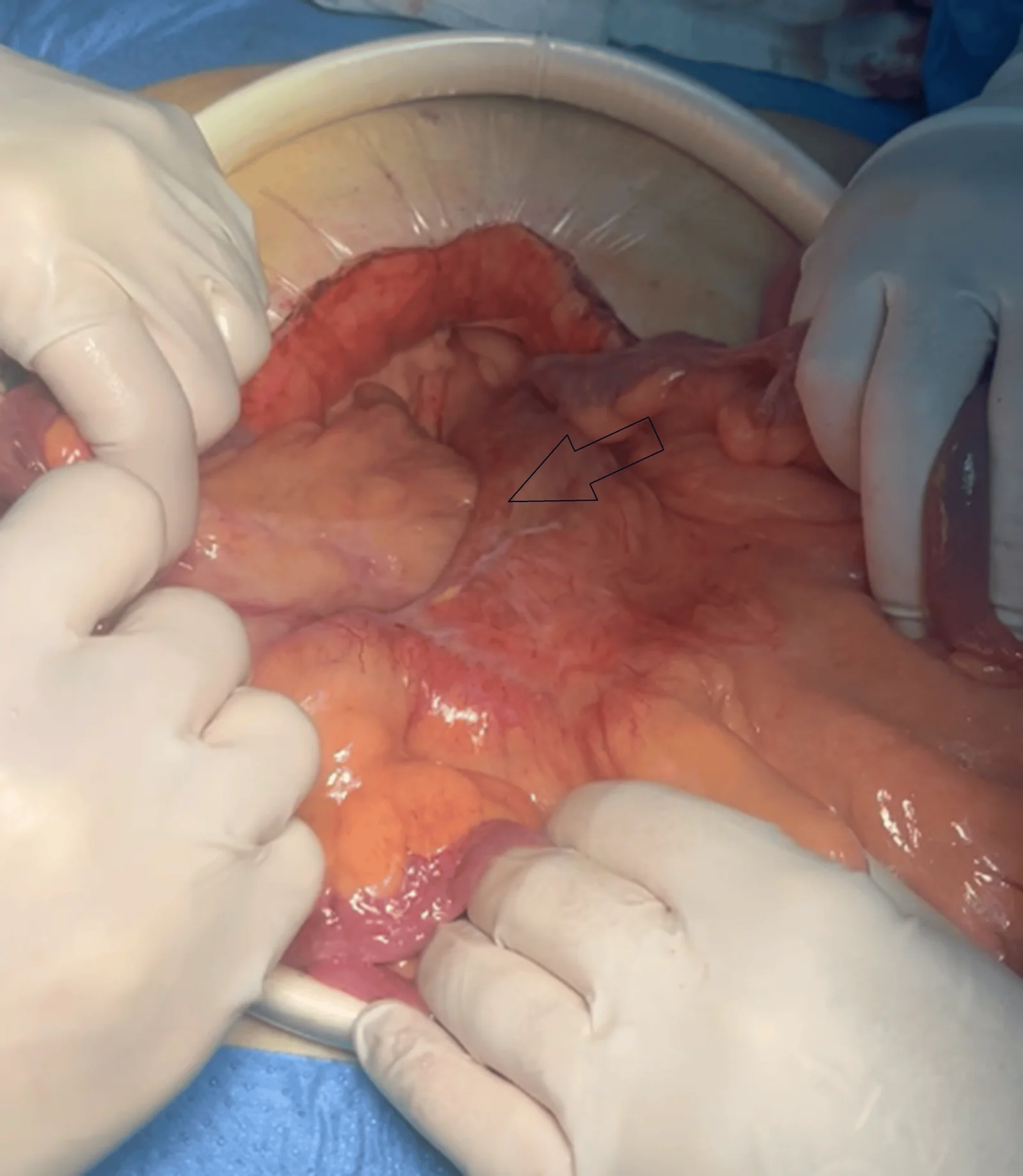
Intraoperative findings and management of right PDH Reduction of herniated bowel loops through the hernial orifice; the hernial defect is clearly visualized (arrow). PDH: Paraduodenal hernia

**Figure 4:**
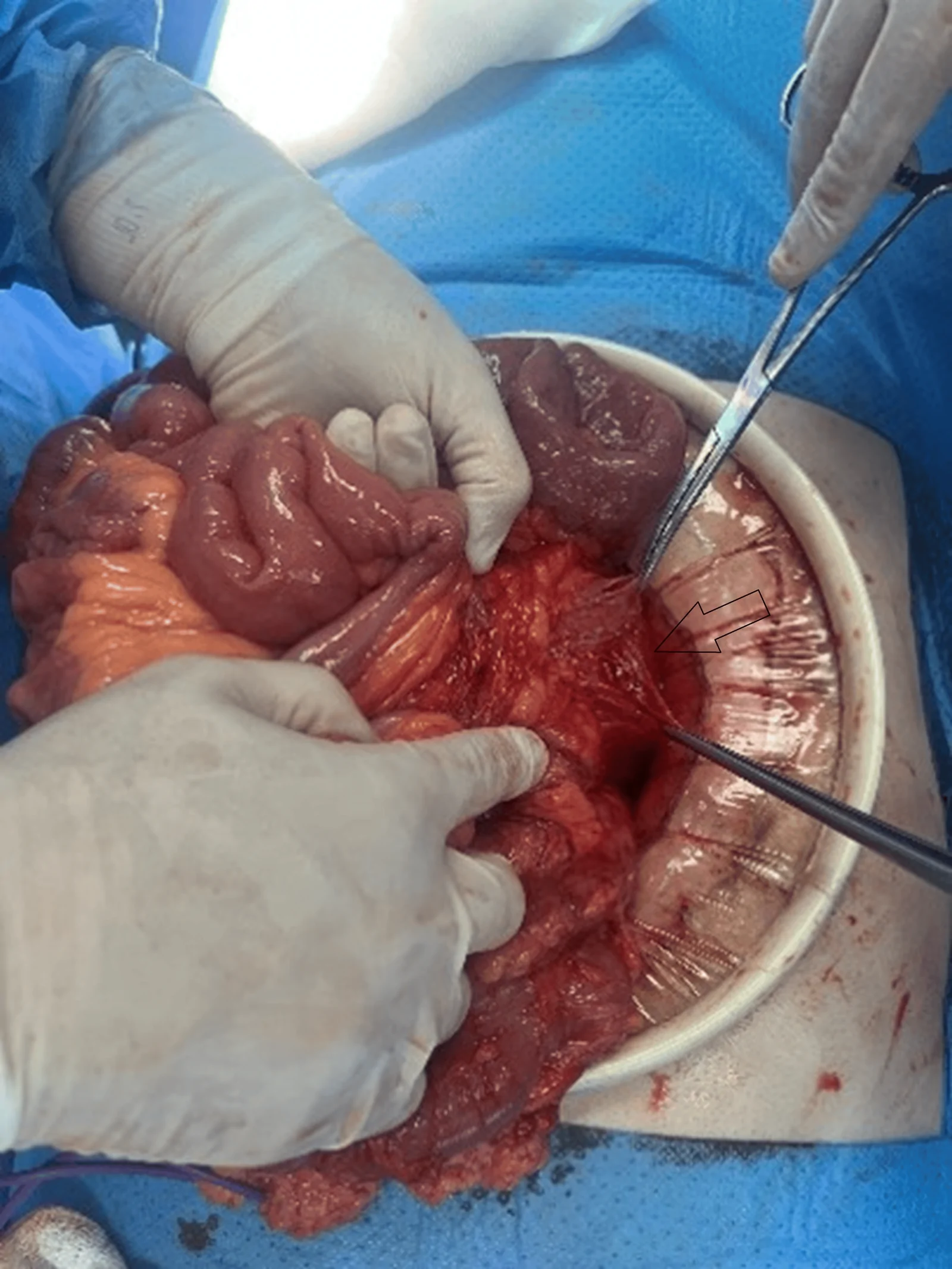
Excision of the hernial sac (arrow) following reduction of the PDH contents PDH: Paraduodenal hernia

To objectively assess bowel viability, an IV dose of indocyanine green (ICG) dye (5 mg in 2 mL) was administered. Within approximately 30 seconds, visualization using a laparoscopic camera system demonstrated rapid and homogeneous green fluorescence across the entire involved bowel segment. This confirmed adequate tissue perfusion and safely supported the decision to preserve the bowel loops. The postoperative course was uneventful, and the patient was discharged on the seventh postoperative day. At the one-month outpatient clinic follow-up, the patient remained completely asymptomatic with no clinical signs of recurrence.

## Discussion

Right PDH is a rare but noteworthy cause of intestinal obstruction, accounting for approximately 1% of small bowel obstruction cases and over 50% of internal hernias reported in the literature [1,3-5]. They can be congenital due to embryonic abnormalities such as midgut malrotation or incomplete mesenteric fusion [4,5] or acquired, often related to trauma or prior abdominal surgeries [4]. Right PDHs are more common in males and are typically diagnosed between the fourth and sixth decades of life [4,5].

Clinical manifestations of PDH vary. While some patients are asymptomatic, others present with nonspecific signs such as abdominal pain, nausea, vomiting, bloating, and constipation [4-6]. The severity of the symptoms correlates with the extent of the obstruction [4,5]. Complications, such as ischemia and necrosis, increase clinical severity and require immediate surgical intervention [3,5]. Chronic postprandial abdominal pain is frequently reported and aligns with the findings of prior studies [5,6].

Diagnosing PDH is challenging due to its nonspecific presentation and low prevalence [3-5]. A CT is the preferred diagnostic modality [2,5,8], showing features like clustered bowel loops and abnormal mesenteric vessel orientation, which are suggestive of an internal hernia [2,5,8]. However, diagnostic accuracy may vary, and laparoscopy may be necessary for a definitive diagnosis in ambiguous cases [5,9].

Surgical intervention is the cornerstone of treatment [4,5,9]. While open surgery has traditionally been the standard, laparoscopic techniques are gaining favor due to their lower invasiveness and faster recovery times [4,5,9]. Surgery aims to reduce the herniated bowel and address the mesenteric defect to prevent recurrence, either by primary closure or by widening the orifice. Primary closure must be performed with extreme caution, as it carries a high risk of iatrogenic injury to the superior mesenteric vessels located along the anterior margin of the defect [4,5,9]. The choice of the surgical approach depends on the patient's clinical condition and the surgeon’s expertise [4,9]. If ischemia or necrosis is present, bowel resection may be necessary [3,5]. Preventing recurrence through meticulous surgical technique is vital for long-term success [4,7,9].

In our case, to avoid catastrophic vascular injury, formal closure of the defect was not attempted. Instead, medial mobilization of the right colon and partial excision of the hernial sac were performed. This technique effectively widens the space and eliminates the narrow anatomical trap, thereby preventing recurrent incarceration without compromising vascular structures.

## Conclusions

Right PDH is a rare and often elusive cause of small bowel obstruction that requires a high index of clinical suspicion. Because clinical manifestations are frequently nonspecific, early utilization of abdominal CT imaging is crucial for an accurate diagnosis and for guiding clinical decisions. Prompt surgical management remains the definitive treatment, essential for preventing life-threatening complications such as bowel strangulation and necrosis. This case highlights specific clinical lessons: the importance of recognizing the atypical imaging features of right PDHs, the value of careful intraoperative assessment of bowel viability (such as with ICG fluorescence), and the necessity of intervening swiftly with a tailored surgical approach to safely resolve the obstruction and optimize patient recovery.
